# Metacognitive Strategies for Developing Complex Geographical Causal Structures—An Interventional Study in the Geography Classroom

**DOI:** 10.3390/ejihpe11020029

**Published:** 2021-05-07

**Authors:** Johannes Heuzeroth, Alexandra Budke

**Affiliations:** Institute for Geography Education, University of Cologne, Gronewaldstraße 2, 50931 Cologne, Germany; alexandra.budke@uni-koeln.de

**Keywords:** metacognitive strategies, systemic thinking, complex geographical causal structures, problem solving, interventional study

## Abstract

This article examines the impact of applied metacognition on the development of geographical causal structures by students in the geography classroom. For that, three different metacognitive strategies were designed: a. action plan, activating meta-knowledge prior to problem-solving and simultaneously visualizing action steps for dealing with the task (A); b. circular thinking (C), a loop-like, question-guided procedure applied during the problem-solving process that supports and controls content-related and linguistic cognition processes; c. reflexion (R), aiming at evaluating the effectivity and efficiency of applied problem-solving heuristics after the problem-solving process and developing strategies for dealing with future tasks. These strategies were statistically tested and assessed as to their effectiveness on the development of complex geographical causal structures via a quasi-experimental pre-posttest design. It can be shown that metacognitive strategies strongly affect students’ creation of causal structures, which depict a multitude of elements and relations at a high degree of interconnectedness, thus enabling a contentually and linguistically coherent representation of system-specific properties of the human–environment system. On the basis of the discussion of the results, it will be demonstrated that metacognitive strategies can provide a significant contribution to initiating systemic thinking-competences and what the implications might be on planning and teaching geography lessons.

## 1. Introduction

The target of the subject geography is to teach and stimulate the comprehension of complex system-relationships [1] (pp. 10–12). The object of these transfer- and acquisition processes, in accordance with an integrative human–environment system approach, are complex systems that represent ecological and social (sub-) systems as well as their effect-relationships [2,3] (p. 85). A series of studies (i.e., [4,5,6,7,8]) have given evidence, however, that students do not succeed at their best, neither contentually nor linguistically, when dealing with complex (multicausal), systemic thinking and developing geographical causal structures (effect-relationships) in the context of complex problem-solving processes in the geography classroom. 

At the same time, systemic thinking or, specifically, geographical causal structures as its smallest component, are the object and result of a complex problem-solving process [7,9]. This problem-solving process is, as the authors suggest, marked by a content-related, a linguistic, and a strategic dimension. Content-relatedly, the problem-solving process requires the identification of a multitude of elements and relationships, the creation of highly interconnected causal relationships between those elements, and thus a comprehensive representation of system-specific properties, structures, and interactions [10,11] (p. 544). This complex representation of system organization and system behaviour [12] (p. 30) is the starting point for solving space-related, complex problems. When verbalizing geographical causal structures, being a representation of system properties, effect-relationships, and problem solutions, high relevance is given to the cognitive function of language (cognitive linguistics; [13]). According to cognitive linguistics, the speaker’s linguistic knowledge changes his or her capacity for, and capability of, complex thinking and its linguistic representation [14,15]. Strategically, dealing with complex problems requires, i.a., effective forms of self-regulation and self-evaluation [16,17]. A promising approach to reduce the challenges with regard to all three dimensions would be the promotion of students’ metacognitive skills through the application of metacognitive strategies and methods in the geography classroom. Metacognition is understood as the bringing to awareness of the declarative (content-related) knowledge, the so-called metaknowledge or metamemory, as well as the awareness of the procedural, i.e., strategic knowledge, or so-called metastrategies [18,19,20,21,22,23]. In the following, metacognitive strategies are understood as methods for their application in the geography classroom. These methods promote the metacognitive and reflexive processes leading to the content-related and linguistic conceptualization of systemic thinking, for the reduction and construction of complex effect–relationships. They furthermore support the required self-regulative organizational processes (i.e., [4,24,25]). The findings of Heuzeroth and Budke [7] (pp. 27–30) in particular suggest that metacognitive strategies for augmenting linguistic awareness and, as a consequence, the capability to verbalize complex geographical causal structures, be increasingly taken into account. 

Yet it has remained mostly unexplained within the research area how those thinking tools and metacognitive methods are to be designed in order to enhance the development of complex causal structures by students in the scope of systemic thinking. What is also still unresolved is, what forms of implementing metacognitive strategies in particular lesson stages are especially effective and efficient. 

This article aims to empirically test the effectiveness of metacognitive strategies for the development of complex, multicausal links by students. Thus, the following research questions will be examined:

F1 What impact does the application of metacognitive strategies have on students’ creation of technically correct geographical multicausal links?

F2 Which of the methods (a) action plan (b) circular thinking and (c) reflection particularly stimulate students’ creation of technically correct geographical multicausal links?

The following hypotheses will be tested in the course of the interventional study:

**Hypothesis** **1** **(H1).** (metacognition and problem solving) *The application of metacognitive strategies when performing problem solving tasks increases the number and correctness of multicausal links in the geography classroom.*

**Hypothesis** **2** **(H2).** (point of use of metacognition) *The point of use of metacognitive strategies, either before or during working on the tasks, affects in different ways the quantity and quality of the technically (contentually) correct multicausal links established by the students.*

A positioning of the topic within the discourse of didactics of geography (Section 2) is followed by a discussion of complex geographical content and its symptomatic, multicausal structure. Multicausal links are part of a multi-layered problem-solving process. Therefore, based on an analysis of the operation of problem solving (Section 3), the difficulties and hindrances of multicausal linking will be examined and the contribution of metacognitive thinking tools and strategies to coping with the “problem space” [26] (p. 141) will be depicted. Derived from that, three different metacognitive strategies (interventions) are presented, which can be applied in developing multicausal relations. Their effectiveness is empirically tested by means of an interventional study and compared with a control group (Section 4). After the presentation of the results (Section 5), central insights into the importance of metacognitive strategies for formulating multicausal links in the geography classroom are critically discussed (Section 6). A brief prospect is eventually given, sketching implications for teaching geography (Section 7). The following materials and worksheets on the metacognitive strategies used in the study are available online at https://geodidaktik.uni-koeln.de/multimedia/metakognitive-strategien-fuer-die-entwicklung-geographischer-kausalstrukturen-im-rahmen-des-denkens-in-mensch-umwelt-systemen (accessed on 7 April 2021).

## 2. Complex Systems in Geography Lessons and Their Multicausal Structure

Human–environment systems and the causal effect–relationships (links) inherent in the system are central content in the subject of geography [1] (pp. 11–12). In the following, the geographic characteristics of complex systems as well as the coherent multicausal geographical causal structures will be explained.

### 2.1. Complex Systems and Causality as Learning Objectives within the Subject Geography

Complex systems, like recycling of reusable resources, and the social, ecological, and economical effects relevant in that scope, are significant content for geography lessons [1] (p. 12). A complex system is hereby made up of elements (e.g., consumers, packaging producers, retailers), the degree of interconnectedness (e.g., system organization, intensity of the use of disposable packaging in everyday life), and the spatio-temporal dynamics (e.g., temporal and spatial development of organizational processes, amount of waste, changes in consumer behaviour) as well as the interaction of the system with its environment (i.e., [27,28] (p. 6)). A starting point for the analysis of a system can be a complex geographical problem [29]; that means, a criteria-based observation that a human–environment system is unstable, not sustainable, or dysfunctional, for example the increase of plastic and packaging waste in the seas. The target can be analyzing the causes for destruction and over-exploitation and investigating the connections between waste generation and pollution in the course of a problem-solving process. The analysis enables students to define an action target (e.g., prohibition of disposable packaging) and develop solution strategies (e.g., using sustainable resources) in order to adjust the system according to one’s goals. Relevant objects for that analysis in complex systems are usually various causalities (or effect-relationships) between the elements of the system or their (multi-) causal interaction (links), respectively.

### 2.2. Characteristics of Multicausal Links in the Geography Lesson as Part of the Geographical Causal Structure

Causality describes a functional interdependence between a cause and an effect. Cause and effect emerge from a direct or indirect link or interaction between system elements [30,31]. The connection entails three aspects: effectiveness, the effective, and their interaction. Effectiveness describes the possibility or the potential that an element can evoke a certain effect or cause an effect (e.g., prohibition of single-use cups). The effective describes the change, i.e., the impacting force that one element has on one or several others (e.g., consumption of coffee in reusable cups). The interaction of cause and effect (e.g., changes in consumers’ behaviour) describes the direction, strength, time, and space of the interaction of the elements (i.a., [32,33]). What needs to be considered is that an effect cannot be placed before a cause in time [34] (p. 290). For Luhmann [35], causality is solely placed within a dynamic (unbalanced) system. Causality is, in addition, always self-referential, which means it may only refer to relations between elements within the same system. The attribution of cause and effect, however, is done from the outside by an observer [36,37] (p. 91). In the geography lesson, causal thinking is an operation by which conclusions on causes (e.g., lack of environmental awareness) are often made looking backwards from observable effects (e.g., plastic waste polluting the environment). The logical reconstruction of a relation takes place afterwards. Those separate operations are then expressed in written or oral language [38] (p. 98). This verbalization of systemic relations is termed geographical causal structure or (multi-) causal link, respectively [8]. 

Based on an integrative human–environment system approach [2,3], multicausal geographical causal structures represent space-related processes, structures, and functions that take into view ecological and social (sub-) systems equally at the same time. With relation to the system–competence model by Mehren et al. [39] (p. 6) [10], multicausal geographical causal structures can be located both in dimension 1 (system organization and system behaviour) and dimension 2 (system-adequate options for actions). Geographical causal structures thereby integrate different competence levels of systemic thinking [11] (p. 544) due to the verbalization of (1) at least three elements and their relationships, as well as (2) a complex degree of interconnectedness, and (3) a differentiated representation of system-specific properties [3] (p. 84) (cf. examples). In the example, monocausal (a) and multicausal or, respectively, complex (b) geographical causal structures on the topic of climate change are depicted.

Examples: 

*a. Monocausal/linear geographical causal structure*:

*Due to the logging of the rain forest, less Co_2_ is stored and the change of the climate accelerates. (Laura, 16 years)*

*b. Multicausal geographical causal structure*:

*Because it is economically highly rewarding and, at the same time, less money is available for environmental protection, more rain forest is logged and cleared. Thereby, less Co_2_ is stored and more emissions are given off. The climate changes thus accelerate. (Timo, 18 years)*

### 2.3. Barriers and Obstacles on the Path to Complex Thinking

From the perspective of psychology (of learning), the simplification of complexity to linear cause–effect relations (reduction strategy) is, among other factors, regarded as a consequence of non-conscious, automatic, and effort-avoiding thinking (i.a. [40] (pp. 698–699), [41,42]; Table 1). Concerning knowledge acquisition and the development of complex thinking skills, scholars refer to the mutual effect of available causal knowledge (“causal models”, [43] (p. 310); Table 1) and mental causal category formation. It is assumed that the attribution of cause or effect of a phenomenon depends on technical and causal pre-knowledge. Likewise, the cognition-triggering task structure as well as the then following forms of information storage in the mental lexicon are of prominent importance when developing complex thinking patterns (i.a. [44,45]; Table 1).

Rinschede and Siegmund postulate, proceeding from Piaget, a developmental-psychological cause and an age-dependent ability of the spatial conceptualization and perception of causality [57] (pp. 55–56) cf. also [52] (p. 17). More complex, logical thinking operations or abstract thinking are thus possible no sooner than at the age of 11 (Euclidian space conception, [48], Table 1). As a central, cross-topical hindrance, Kaminske [52] points out the “coincidence” of too many elements that significantly affect the identification of systemic effect-relationships. The mutual interference and feedback between system elements impede a factually logical identification of cause and effect, including the assignment of effect direction and type [52] (p. 21; Table 1). Mambrey et al. [30] (pp. 15–17) analyse the often-missing factual understanding of students concerning causal relations and system properties, and conclude that there exist domain-specific hindrances for complexity (Table 1). Mehren et al. [39] (p. 4) point to a “close-environment focus” (“Nahbereichsspezialisierung”), i.e., the attempts of humans to limit what is relevant to the sensually perceptible area, based on an emotional, affective load that subsequently prevents complex, multicausal linking (Table 1). Heuzeroth and Budke [7] (p. 20) found in their research that the application of linguistic scaffolds or multilingual learning settings in the geography lesson positively affect the contentual and linguistic quality of causal relations and the number of causal links established by students (Table 1). At the same time, they observed, in coherence with many other authors’ findings (i.a. [52,58,59]), that expressing multicausal links poses a particular difficulty for students. 

## 3. Problem Solving and Metacognitive Knowledge and Strategies—Core Aspects of Establishing Multicausal Links

The development of multicausal links in the lesson is often part or the result of a complex problem-solving process. In the following, central characteristics of problem solving, in particular control and monitoring of cognitive activities and actions, in short metacognition, will be discussed and their effects on the development of multicausal links depicted.

### 3.1. Features of Complex Problems in Geography Lessons

In the subject geography, students’ problem-solving competence is to be initiated by means of complex problems [1] (pp. 5–6). The objects of this problem-solving competence are the interdependencies and effect-relationships within the system earth–human as well as their natural geographic and human geographic sub-systems [1] (pp. 10–12). In contrast to analytical problems, where all information relevant to the solution is entailed in the problem situation or can be accessed by deductive reasoning [24,60], complex problems require the exploration of the problem situation by an examination of its elements as well as the effect-relationships of the system [60] (pp. 18–19). A problem emerges from the presence of targets (e.g., environmental protection) and defines therefore a desired state (target state). Complex problems are marked by their dynamics, a multitude of targets, interconnectedness, non-transparency, and complexity [61] (p. 155) [38] (pp. 58–59). Normally, complex problems are “poorly defined situations” [61], that means an evaluation of the validity of the solution concept appears difficult. Referring to geographical relations, Budke [29] (pp. 25–27) differentiates three types of problems: (a) understanding- und knowledge gaps, (b) contradictions, and (c) complexity. The problem’s solution is thus defined by (a) resolving the lack of understanding, (b) obtaining evaluative certainty, and (c) the comprehension of complex interrelationships. 

Newell and Simon name the path from the initial state (problem) to the target state (problem solution), via various intermediary states where a multitude of thinking operations are conducted and the knowledge base changes, the “problem space” [26] (p. 21). The thinking operation required to deal with that problem space is called problem-solving thinking [38,61]. Since routine actions are not available [61] (p. 139), problem-solving thinking is a creative process of heuristically searching for solution strategies [62] (p. 326). These heuristics for closing the gap between the status quo and the target state demand the application of transformation knowledge, creativity, metacognitive skills, and, finally, a reconstruction of knowledge bases [63] (pp. 181–191). This reconstruction represents the contentual transformation of the actual state (problem question, information in the material, fragility of the eco-system) into a desired target state (reduction of unwanted phenomena). To what extent metacognition may constructively contribute to dealing with the problem space and thus promote thinking in complex geographical causal structures will be examined below.

### 3.2. Metacognition—Knowing about Knowledge and One’s Own Strategies

Flavell [18,19] differentiates metacognition into knowledge of strategies and knowledge on one side and, on the other, into surveillance of one’s own uses of strategies, i.e., the knowledge of dealing with cognitive challenges (e.g., problem-solving), as well as controlling and monitoring of one’s own activities. Metacognitive knowledge (meta-memory) comprises intrapersonal cognitive structures and processes that refer to the knowledge about one’s own thinking and cognitive acquisition processes (declarative dimension, i.a. [64,65]). Metamemory refers to our knowledge and awareness of our own memory processes. In this case, knowledge means self-knowledge about our memory processes [66] (p. 8) [67]. Knowing about the planning and controlling of one’s own thinking and acting while preparing and performing one’s actions constitutes the procedural dimension [68] (p. 130), [20]. The knowledge about one’s own knowledge and thinking potentials is referred to as person variable (self-knowledge). Knowledge about the demands entailed in a problem or task are specified by Flavell [18] (p. 907) as problem variables (knowledge about tasks). Knowledge about strategies and when what strategy can be applied effectively is termed strategy variable (strategic knowledge; [18] (p. 907), [69] (pp. 220–222)). Hasselhorn [20] (p. 42; Table 2) differentiates five sub-categories of metacognition: systemic knowledge (knowledge about strategies, learning requirements and one’s own cognitive activities), epistemic knowledge (knowledge of one’s own knowledge), executive processes (planning, controlling, monitoring), sensitivity (experience, intuition), and metacognitive experiences (knowledge of one’s own emotions and learning experiences). The sheer multitude of metacognition and the various effects on cognition processes allows the assumption that there is a large effect on the development of multicausal links, which will be further explained in the following section, with particular regard to the development of metacognitive methods. 

### 3.3. Difficulties in Problem Solving and the Creation of Multicausal Links—The Possible Value of Metacognitive Strategies

The development of multicausal links by students in the geography lesson can potentially be stimulated through metacognitive strategies and methods, since these affect all phases of the problem-solving process, such as target identification, self-instruction, self-evaluation, and contextualization [70] (pp. 102–104), [71] (Table 2). Before dealing with the task itself, respective strategies may support students in sorting out the contentual and situative problem contexts (transparency) [72] (p. 684), e.g., by activating the domain-specific prior knowledge, helping to understand the task, and supporting (encoding, [62] (p. 322), [42] (p. 342)) the identification of the target (target state, see above). Metacognitive strategies might support students to understand the dynamics, multitude of targets, interconnectedness, non-transparency, and complexity of problems. Metacognitive strategies may have very positive effects on emotions (e.g., subjective attitudes towards, and experiences with, the topic) and the motivation of students [73]. The resulting attention (-control) or raised concentration capacity [61] (pp. 138–141) can crucially affect the successful mastering of the problem solving process and thus the creation of multicausal links [74].

**Table 2:** Selected characteristics and effects of metacognitive strategies on learning and problem solving.

Reference	Characteristics/Recommendations/Findings on Metacognition
[18,19]	The knowledge of effective storage and recalling strategies, knowledge about these strategies, knowledge on what strategy to use for what challenge, and the monitoring during its application.
[16,75]	Control, surveillance, and monitoring processes as fundamental features of efficient thinking. They can be promoted through: I. Prior to working on the task: Recognizing and checking the cognitive requirements and the identification of internal action routines or targeted strategies as well as planning task handling when defining distinct action steps; II. While working on the task: permanent surveillance, coordination, and corrections of task solving and maintaining awareness and motivation; III. After finishing work on the task: Evaluation and inspection of target accomplishment and its quality.
[64,76,77]	Metacognition as a construct consists of a metamemory and metacognitive (executive) strategies. The metamemory positively affects the performance with memory-based tasks. The presence and application of executive components proved to raise the performance of work on tasks.
[20]	Differentiation of metacognition into 5 sub-categories: 1. Systemic knowledge, 2. Epistemic knowledge, 3. Executive processes (monitoring) and planning own learning processes, 4. Sensitivity for the potentials of cognitive activities, 5. Metacognitive experiences concerning one’s own cognitive activity.
[78,79]	The sensitization for misconceptions of one’s own subjective performance by means of metacognitive strategies and feedback can positively affect students’ performance and learning success.
[31]	Explicit use of a technical system language and technical system properties by the teacher during the teaching and learning activities enhance students’ metacognitive thinking processes and enable them to perform a skill transfer to, and application within, other contexts.
[80]	(1) Embedding of metacognitive instruction into the subject matter of teaching in order to secure compatibility, (2) information of learners on the usefulness of metacognitive activities in order to motivate them to undergo the initially additional effort, and (3) extension of the training in order to secure the smooth and constant application of the metacognitive activity.
[74]	Metacognitive monitoring: strategies are applied to monitor learning behavior, having a positive effect on learners’ success.
[81]	Metacognitive competences and the precision of task handling (performance) as well as matching (correctness) of task relations have a great mutual influence.

In the course of the problem solving process, metacognitive strategies may lead to an effective monitoring and control of cognition and strategy use (executive component; [16]; Table 2). Thus, existing knowledge structures and strategic resources for the process of dealing with, and the solution of, a problem are mobilized (means-goal-analysis; [17] (pp. 338–339). The mobilized knowledge structures and resources may presumably increase students’ abilities to choose, categorize, and combine (selection) available information (system elements, causal relationships) according to their relevance. In this way, problem-related relevant system elements and their causal structure are expected to be identified sooner. Once single system elements have been identified, metacognitive strategies supposedly support the recognition of target-related possibilities of combining systemic causal links and the direction and strength of the interdependencies. This recognition would be a result of the (metacognitive) circular comparison of existing internal causality concepts and models, as well as possible problem solving variants that contribute to the goal of reducing the gap between status quo and target status [82] (pp. 212–214) [72].

The targeted application of metacognitive strategies supports the development of multicausal links through the organization and transformation of knowledge [71]. This happens owing to a structuring of the problem-solving process as well as the activation of and the access to students’ conceptional knowledge. 

Key aspects are the strategies accessible to learners which allow them to make a choice from the given information content and solution options [42]. That choice takes place based on certain subjective heuristics or, alternatively, clearly defined acting and thinking patterns. During the process of metacognition, these heuristics get activated more easily and repeatedly adjusted in relation to the problem’s demands (multicausal links) in connection with the technical and linguistic resources available to the students [18] (p. 908) (Table 2). They might, as a consequence, be more capable of monitoring the development of complex multicausal links and of verifying or rectifying the result, i.e., the linguistically correct representation of (geographical) content relationships [81] (Table 2). So far, it has remained mostly unexplored how the triad effective in the classroom, consisting of motivation, cognition, and metacognition [83], ought to be designed in order to successfully develop complex geographical causal structures in the geography lesson. In particular, the effectiveness of metacognitive strategies on the creation of multicausal links in the context of thinking in human–environment relationships is as of yet mostly unexplained.

## 4. Methods/Survey Design

**Figure 1:**
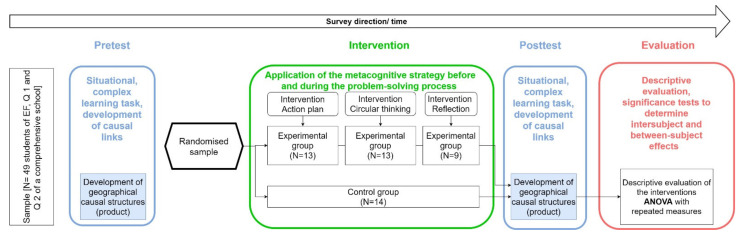
Survey design (own representation).

The research hypotheses and questions (cf. Section 1) were examined via an explanative study with a quasi-experimental randomized pre-posttest design [84] (pp. 213–214) [85] (p. 258) (Figure 1). 

The depicted survey design (cf. Figure 1) was applied in December 2020 in three senior classes (The senior level leading to the Abitur (higher education entrance qualification) in North Rhine-Westphalia consists of a one-year introduction stage and a two-year qualification stage) at a Gesamtschule (A “Gesamtschule” (comprehensive school) is a German school type that offers different educational programs within a single school) in the German state of North Rhine-Westphalia (NRW). Due to the general schools’ closure from December 2020, in connection with the SARS-CoV2 pandemic, the posttest had to be conducted via distant-learning, i.e., digitally. This circumstance brought about a higher sample mortality. The sample size amounted originally to 66 students (male: n = 29; female: n = 37) aged between 16 and 20. At the survey’s end, data of n = 49 study subjects (male: n = 20; female: n = 29) were available. According to our a priori test strength analysis (G-Power, 1 − β = 0.80, α = 0.05, f = 0.25, [84] (p. 841)), a sample size of n = 48 ought to provide significant and reliable statistical effects. The randomization, that secured an equal distribution of characteristics of the survey group and the control group, was done by means of the “matched sample” method, taking into account the results of the pretest, grades in the subjects mathematics, German, and geography, as well as age and the gender of the survey subjects [84] (pp. 200–201), [86] (p. 140). Additionally, the confounding variable of language or, respectively, the linguistic barriers at expressing causal links, were to be disabled by using a linguistic support tool which was made available to all groups. The statistical correlations of the effect of metacognitive strategies on the number and correctness of multicausal causal structures were tested regarding the individual improvement in creating multicausal links (within-subject effects) and regarding the differences in the effectiveness of the applied strategies (between-subject effects). The results were encoded and analysed via repeated measures ANOVA in SPSS 27 [87] (p. 997).

### 4.1. Expression of Thinking in Complex Human–Environment Relationships—Geographical and Linguistic Features of Multicausal Geographical Causal Structures

Human–environment systems are marked by various causal relationships (Section 2.1 and Section 2.2). Those causal relationships have to be verbalized by students in the scope of systemic thinking in the classroom (Section 2.3 and Section 3.3). The verbalization of causality in the geography classroom happens by means of geographical causal structures. On the basis of complexity (quality: mono- or multicausal) and accuracy of the verbalized causal structures (dependent variable), the effectiveness of metacognitive strategies (explanatory variable) is to be measured (Figure 1). According to Heuzeroth and Budke [7] (pp. 4–5), a geographical causal structure consists of content-related geographical technical terms (e.g. waste, disposable packaging, resources) or phrases that are composed of nouns and adjectives. These denote the system elements and their spatial positioning. Verbal constructions (such as “reduce”, “determine”) or adverbial phrases (e.g., based on the high consumption…”) express the time, direction, and strength of the causal interdependency. Verbal constructions thus qualify the relationships between the geographical elements [55] (pp. 256–259), [88] (p. 108). The use of adverbial sentences by means of a main-subordinate clause construction allows marking cause and effect on the sentence level. A peculiarity of the multicausal link is the (potential) connection of several sentences to express a complex effect relationship. There exist different sentence types with specific (causal) characteristics [89] (p. 328).

**Figure 2:**
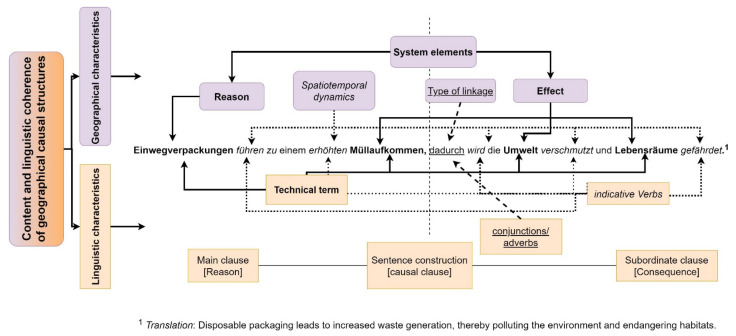
Geographical and linguistic features of multicausal links (own representation).

The example sentence (Figure 2) is a causal sentence. The function of the subordinate clause is defining a consequence for the reason in the main clause (“carrier clause”) [90] (p. 378). The main and subordinate clause are preceded by a (causal) conjunction, subjunction, or adverb, for example “as” and “because”, “since”, or “therefore” (in the example Figure 1 with the adverb “thereby”/“dadurch”). These express an interdependency between cause and effect. They function as causal markers on a lexical level [55] (pp. 258–262). Multicausal links are characterized by the presence of an effect-relationship between at least two causes and an effect or, respectively, a cause with at least two effects. Multicausality differs from linear (effect-) causal chains by the number of interlinked system elements. On the one hand, system elements of different hierarchical levels can be linked multicausally. On the other hand, different degrees of interconnectedness (e.g., feedbacks) can be represented by geographical causal structures. Moreover, multicausal geographical structures may relate several spatial entities and temporal process dimensions (spatio-temporal dynamics, i.a. [91] (p. 45), [7] (pp. 5–8)). A successful (multi-) causal linkage in the geographical context is contentually and linguistically coherent. Linguistic coherence can be spoken of if spatial, temporal, and causal encoding allows a linguistic allocation of causes and effects [55] (p. 280). Content coherence means that the linguistic allocation of a cause to an effect corresponds with the current geographical or subject-specific knowledge [7] (p. 7), [8] (p. 13). 

The dependent variable “multicausal link”, thus the effect of metacognition, became manifest in the respective quantity and accuracy (testing according to the model by Heuzeroth and Budke [8] (p. 25)) of multicausal links in the results of the pre and posttests. Based on the changes in numbers as well as the changed portion of the contentually and linguistically correctly (accuracy) produced geographical causal structures, the effects of the three different interventions (a–c) were tested. Contentually and linguistically correct multicausal links were furthermore tested as to their topical content-relatedness to the problem question (matching). Criteria for topical matching were the applied technical terms expressing recognized system elements as well as topical effect relationships represented by indicative verbs and conjunctions. Additionally, the individually created geographical causal structures were analysed concerning the number of used items of spatial information and their changes before and after conducting the interventions.

### 4.2. Methods of Promoting Metacognitive Strategies

Owing to the interconnectedness of the individual aspects of metacognition, it is difficult to measure them in an isolated way [20] (p. 43). As a consequence, three metacognitive interventions have been developed: (a) action plan (A); (b) circular thinking (C); (c) reflection (R), and are applied in one experimental group each (all material on the interventions can be found under [92]). They will be explained in brief below.

(a) Action plan (A)

The metacognitive knowledge and strategies are to be activated, organized, and verbalized by means of an action plan and thus be made available for the task-solving process [93] (pp. 79–83). The method’s targets are increasing problem comprehension, activating prior knowledge, and bringing to mind action steps during the problem-solving process. It thus aims to support structured proceeding when creating and evaluating geographical causal structures. Impulse control and pausing, hence inhibition of actions, are the first step towards awareness and thus further approaching the problem solution and thereby the development of multicausal links [94]. Identifying a problem, bringing to mind the available skills [82] (p. 215), weighing up different solution paths, and applying them in a controlled manner promise to be a productive element to successfully manage the problem-solving process and develop contentually and linguistically correct multicausal links as a consequence [20] (p. 35). By means of the action plan [92], knowledge regarding one’s own thinking proficiency and thinking strategies is supposed to be activated, which is subsequently available for various encoding processes (i.a., problem comprehension (“Problemverständnis”); [65] (pp. 380–383)). The plan’s construction promotes the development of individual, yet problem/solution-related criteria for reducing content complexity, reduces the risk of overload by informatory diversity (“cognitive load theory”, [95,96]), and enables the activation of contentual and strategic prior knowledge and self-regulating processes of planning and acting (volitional phase; [73] (pp. 138–142)). It involves “problem-solving though application of strategies” [63] (p. 184) and supports learners in the stage of orientation and planning of the problem-solving process [97] (pp. 62–65).

(b) Circular thinking (C)

The second intervention is to support the effectivity of the problem-solving process through question-guided, circular thinking [92] and thus promote the creation of multicausal links. Circularity thereby means a loop-like, question-guided procedure in five steps: 1. Recognizing causes/effects; 2. Defining effect-relationships; 3. Verifying the matching of effect-relationships to the problem question; 4. Verbalizing effect-relationships; 5. Evaluating the causal and linguistic accuracy of the effect-relationships. By stimulating circular thinking strategies, problem-solving potentials are to be revealed and the appropriate form of problem-solving for particular tasks or targets can be found [94]. This interlinked and dynamic thinking [27] (p. 23) requires a feedbacking, circular or spiral-shaped matching-process between the identification of the elements, of the interrelationships, and thus the potentials of the system state [82] (pp. 212–213). The approach of circular questioning and thinking borrows from systemic therapy [98,99] (p. 118) and hermeneutic questioning [100] (pp. 478–480), [101] (p. 230). It concedes students a conscious (observing) distance to the learning object. In this way, the information gain and analysis of patterns, processes, and causal interdependencies are structured and enhanced, and what is more, the student’s own knowledge resources are activated and visualized [99] (p. 141). Present questions (“Gegenwartsfragen”) and reality-constructing questions (“Wirklichkeitskonstruktionsfragen”) come to use. They help to better comprehend the context of the problem, adjust the perspective on the system, and identify new forms of causal links (interactional relations) [99] (pp. 145–146). The intervention supports learners’ monitoring processes in the executive stage of problem solving [97] (pp. 62–65) by activating individual action-control strategies and self-regulating mechanisms [73] (p. 149).

(c) Metacognitive Reflection (R)

The effect of metacognitive reflection stages is tested through an individual evaluation of the pretest results in connection with students’ specific thought impulses and poses the third intervention. The intervention consists of four central aspects: 1. An individual content-related assessment of the quality of the target accomplishment/problem solution; 2. Self-evaluation of contentment with one’s own results; 3. Reflection on the applied strategies leading to the problem’s solution; 4. Formulation of targets and steps to improve future handling of tasks [92]. The intervention reflection aims at identifying and elaborating the effectivity and efficiency of the problem-solving heuristics applied and thus support the development of metacognitive competences (i.a., [23] (p. 5), [75] (pp. 142–144)). Converting successful heuristics into strategies plays a central role in developing metacognitive knowledge [60] (pp. 18–19). This conversion is the result of reflecting and testing the solution and the approach to the problem based on the criteria: (a) accuracy and (b) efficiency [97] (p. 70). This process is supported by a question-guided analysis with sample solutions that are related to students’ own solutions.

**Figure 3:**
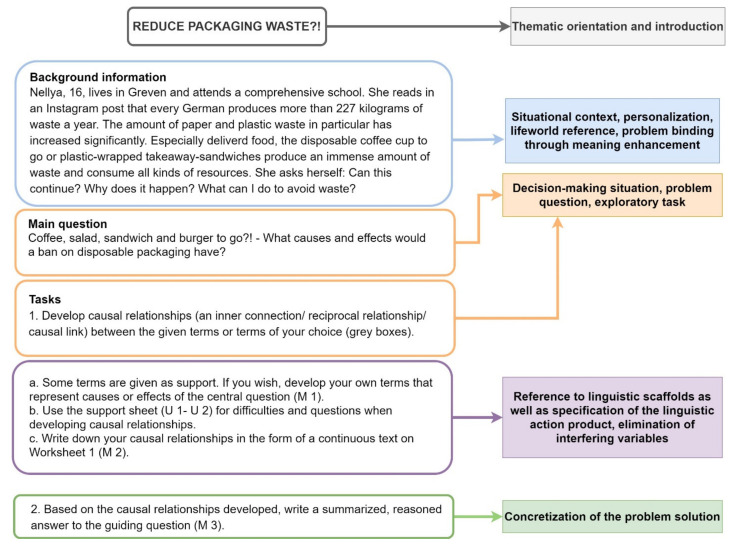
Task structure with pre- and posttests (here: topic of the pretest, own representation).

In accordance with the goal-setting theory, task definitions for all interventions were formulated in a specific, precise, realistic, and challenging way, so that accomplishing the target could be concretely operationalized by the students [102] (pp. 222–223) (Figure 3). The tasks were set exploratively [38] (p. 198). The target binding, however, was increased by embedding the task into a situative decision context (“situative learning”) [103] (p. 283) in order to augment the student’s sense of purpose of the task [73] (p. 138). The respective material was piloted in a class of the introductory stage. Thereby, the task formulation and description of the situation were specified, and the layout arranged so as to be easier to read.

## 5. Results

The results of the pre and posttest are first descriptively presented. Thereafter, the hypotheses are analytically tested based on the obtained quantitative data, via SPSS 27 and repeated measures ANOVA.

### 5.1. Descriptive Account of the Data from Pre- and Posttests

**Figure 4:**
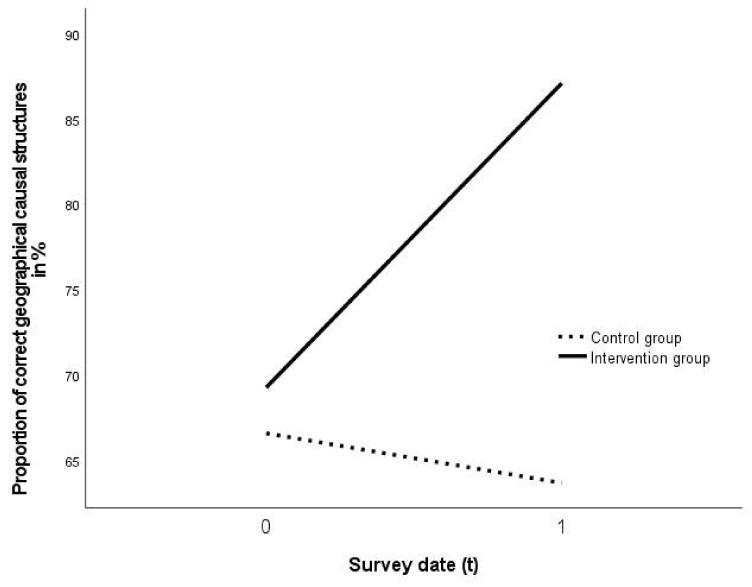
Effect of the intervention on the accuracy of geographical causal structures (n = 49) (own representation).

In total, for n = 49 students (age: *M* = 17.02, *SD* = 0.878) prior to the intervention (t_0_) 260 (*M* = 5.31) and after the intervention (t_1_) 276 (*M* = 5.63), causal links were detected (for examples, cf. Figure 4). According to these figures, a significant increase in the number of causal links occurred. In t_0_ n = 111 (*M* = 2.27) and in t_1_ n = 185 (*M* = 3.78), multicausal links were created, representing an increase by 66.67%. Thus, not only the number of causal, but also that of multicausal links was augmented after the intervention. Of the links formulated by students, in t_0_ = 177 (68.08%) and in t_1_ = 228 (82.61%) were matching the topic, as well as contentually and linguistically correct (Table 3; Section 4.1).

In order to illustrate the collection and evaluation of geographical causal structures at pre and posttest, two examples are given in the following:

(I) There would be fewer orders, which is why less exhaust fumes could be detected in the environment (Paul, 18 years).

The student’s answer (I) stems from the pretest (Figure 1). It represents an incorrect multicausal causal structure. In spite of identifying topically relevant system elements (orders, environment, exhaust fumes) and the use of a causal clause (here: main- subordinate clause structure), the used construction (which is why) is contentually and linguistically inaccurate. The indicative verbs (be … fewer; could be detected), in connection with the conjunction, represent no clearly identifiable spatio-temporal dynamics of the described effect-relationship. The geographical causal structure has not been developed correctly.

(II) Because the online market has a greater choice, people purchase there and the retail shops make less profit (Lena, 17 years).

The answer (II) stems from the posttest (Figure 1) from the intervention group circular thinking (C; Section 4.1). It represents a correct multicausal causal structure, in spite of minor linguistic shortcomings. This is effected by the correct identification of the topically relevant system elements (online market, choice, people, retail shops, profits) and the application of a causal clause (here: subordinate-main clause structure), which is lead in by the correct conjunction (because). Despite the fact that the indicative verbs (has; purchase … there; make less) are linguistically not completely accurate, the spatio-temporal dynamics of the effect relationship can be extrapolated. The geographical causal structure has been developed correctly. 

It becomes evident that the portion of higher-quality links by students has greatly increased after the application of metacognitive strategies in the course of the intervention. The decrease in the share of the correct causal links in the control group (−2.91%), compared with the increase among the intervention groups (H = 31.70%; C = 25.79%; R = 17.86%), highlights the effectiveness of the tested metacognitive strategies. Both interventions for promoting metacognitive thinking: action plan (A), and circular thinking (C), have each lead to an increase in the number of causal links and their content-related matching with the complex problem-solving task (Table 3). As to the intervention reflection (R), the total number of links sank (Table 3, t_0_: *M* = 6.44, t_1_: *M* = 6.22), although here the highest initial level was found in t0. A statistical effect of age and gender on the number and correctness of multicausal links was not observed (age: *F*(4.000, 44.000) = 0.194, *p* = 0.941; gender: *F*(1.000, 47.000) = 2.013, *p* = 0.163).

### 5.2. Effect of Metacognitive Strategies in the Development of (Multi-) Causal Links

In this chapter, both research hypotheses (a and b) will be tested based on the obtained data. The statistical analysis of the effect of the interventions on the creation of complex geographical causal structures yielded the following results.

(a) H1 (metacognition and problem solving) The application of metacognitive strategies when performing problem solving tasks increases the number and correctness of multicausal links in the geography classroom.

Founded on the evaluation of the aggregated data of the intervention groups (n = 35) in relation to the control group (n = 14), with regard to the development of the number of geographical causal structures, a statistically significant interdependency was determined *F*(1.000, 47.000) = 6.035, *p* = 0.0178, partial η^2^ = 0.113798 (A repeated measures ANOVA with a Greenhouse-Geisser correction determined that mean performance levels showed a not statistically significant difference between measurements) (Table 3; Figure 4). The portion of correct causal structures rose from 69.69% to 86.96%, whereas the portion of correct causal structures decreased from 66.57% to 63.66% among the control group (Figure 4).

**Figure 5:**
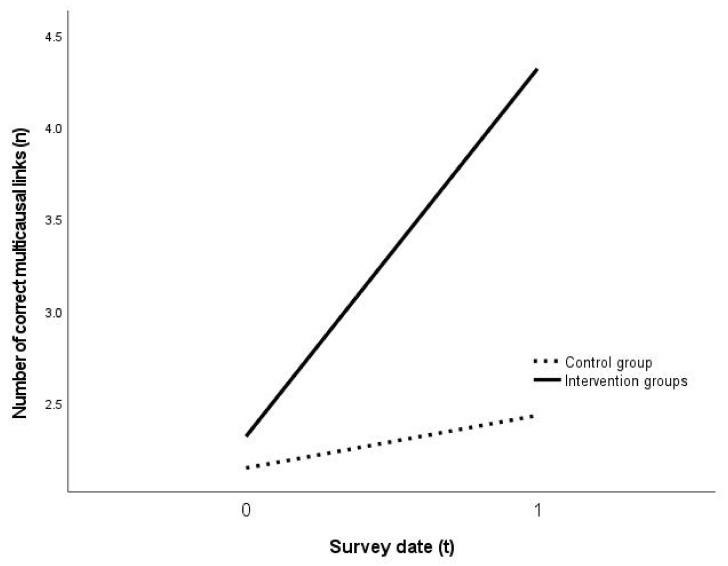
Effect of the intervention on the number of geographical causal structures (own representation).

The analysis of the aspect of accuracy of the created multicausal links (Table 4, Figure 5) also revealed a statistically significant correlation: *F*(1.000, 47.000) = 5.553, *p* = 0.0227, partial η^2^ = 0.105667. The statistical effect of the intervention on the accuracy of the students’ multicausal links amounts to f = 0.32, and on the number f = 0.36, thus representing a medium effect with a tendency towards a large effect size [104] (pp. 287–289). 

Founded on the data, hypotheses H1 can be confirmed on the basis of the available sample. The applied metacognitive strategies are evidently highly effective on the problem-solving process and obviously support the construction of multicausal links by students in the geography classroom (Figure 4 and Figure 5, Table 4).

Whether the timing of the application of metacognitive strategies exhibits differing effectiveness on the creation of complex geographical causal structures will be shown in the following by testing the second research hypothesis (b).

(b) H2 (point of use of metacognition) The point of use of metacognitive strategies, either before or during working on the tasks, affects in different ways the quantity and quality of the technically (contentually) correct multicausal links established by the students.

**Figure 6:**
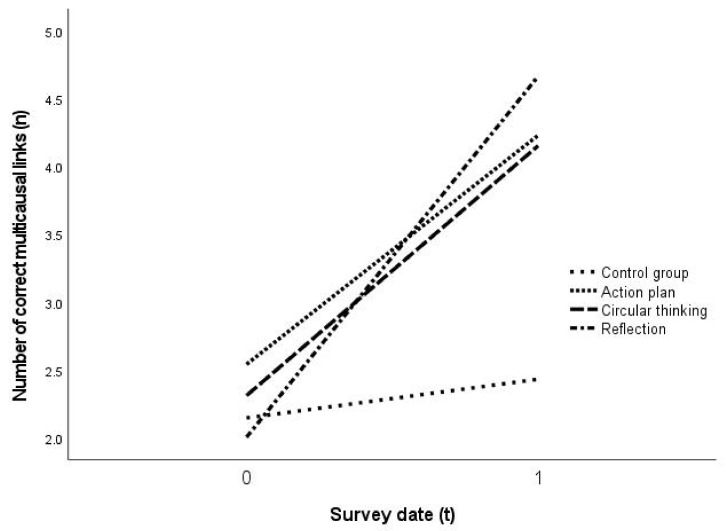
Number of multicausal links in t_0_ and t_1_ according to groups (own representation).

The individual observation of the control group and the three intervention groups (n = 49) revealed a significant main effect on the time, i.e., solely based on the repeated application of the intervention with *F*(1.000, 45.000) = 25.238, *p* < 0.001, partial η^2^ = 0.359320, as well as a very large effect of f = 0.560 [104] (p. 287) (Table 4; Figure 6). There was, however, no statistically significant effect observable between the allocation to a particular intervention group and the number and accuracy of multicausal links *F*(3.000, 45.000) = 2346, *p* = 0.085 (Figure 6). Therefore, a qualitative differentiation of the individual interventions regarding their effectiveness on the development of multicausal geographical causal structures based on the point of their application in the problem-solving process and the design of the intervention could not be statistically proven on the basis of the present data *F*(3.000, 45.000) = 2.346, *p* = 0.0854, partial η^2^ = 0.135245. The effects of the change in number and accuracy of the multicausal links formulated by the students were similarly high in all three intervention groups (Table 4 and Figure 6). The reduction of monocausal links by the intervention group reflection (Table 4) in particular, and the increased effect on the development of multicausal links (increase of multicausal links: *M* t_0_ = 2 on t_1_ = 4.67, Table 4), have to be highlighted. What similarly has to be pointed out is the high increase rate in the proportional accuracy of the multicausal links by the intervention group action plan (Table 4).

## 6. Discussion

Based on the present study, it can be concluded that the application of metacognitive strategies and methods has a large effect on the construction of contenually correct, and on the number of multicausal geographical, causal links (Table 4; Figure 6). Accordingly, metacognition leads to a qualitatively and quantitatively improved creation of multicausal geographical causal structures when solving complex tasks (Table 4, Figure 5 and Figure 6). Additionally, it becomes clear how important the implementation of metacognitive strategies of thinking and acting might be for learning-effective geography lessons, in particular concerning the promotion and the establishment of systemic thinking competences (i.a. [10,105] (p. 4)). 

As a consequence of the study, it can be assumed that metacognitive control and monitoring processes raise the task-related and topical accuracy. This confirms findings by Vuorre and Metcalfe [81] (p. 19) and Dunlovsky et al. [74] (pp. 23–27) on the connection of metacognitive competences and the precision or matching of the task processing or learning success, respectively. It nevertheless remains unresolved what metacognitive strategies or methods are particularly suited for initiating individual system competences [27] (pp. 25–32), [106] (pp. 43–44). Especially the impact of metacognition on the problem-solving process and the domain-specific acquisition of a geographical, systemic expertise [107] (pp. 56–59) becomes evident and affirmed through the study. Previous results, be it regarding the effect of metacognitive competences on knowledge acquisition and application in the problem-solving process [51] (p. 20), or on the comprehension of the problem situation and weighing up different possible solutions [108] (pp. 3–6), applying different solution heuristics [58] and a targeted evaluation of the solution result [72] (pp. 682–685), [105] (pp. 110–116) can be backed by the findings of this study. 

It was shown that the developed methods action plan, circular thinking, and reflection promote the creation of contentually correct (geographical) multicausal causal structures. A qualitative differentiation, which method affected the problem-related control and monitoring of students’ individual cognitive activities, could yet not be made [16] (Table 4, Figure 6). The method “action plan” (A) very successfully promotes metacognitive strategies leading to a self-activation of linguistic and contentual prior knowledge (metamemory), the comprehension of tasks and problems, as well as an improved self-organization (metastrategies) by students [82,93,97]. The method “circular thinking” (C) predominantly supports the analysis of system elements and their effect-relationships, and also their synthesis with relation to the problem [11]. At the same time, the conscious control of attention strengthens the use of strategies in the problem space [80]. The method “reflexion” (R) stimulates particularly the awareness of one’s own metastrategies and the assessment of their appropriateness [73] (pp. 138–142). The findings of this study furthermore suggest that the development of new problem-solving heuristics for future problems is promoted [109].

Age and gender did, in the present study, not have a verifiable effect on the ability to create causal links, which, however, can presumably be explained by the (homogenous) developmental–psychological age structure of the sample [48]. 

The implementation of a linguistic scaffold to eliminate the confounding variable “lack of linguistic means” facilitated students’ linguistic representation of complex, multicausal effect relationships and thereby the development of multicausal links [7,8,54]. What becomes evident at this point is that systemic thinking requires a specific linguistic knowledge. Linking technical knowledge and technical language skills is an important factor for complex thinking processes. The (cognitive) function of language ought to be more taken into account by the respective implementation of linguistic scaffolds, thus supporting the findings of Heuzeroth and Budke [7] (pp. 27–30).

The very large effect of metacognitive multicausal links might, as a constriction, be traced back to the two measurement time points and the panel effect that are potentially connected hitherto [86] (p. 145). In order to confirm the results of this survey, a long-term study is recommended, entailing several repeated measurements to minimize the temporal effect, on one side, and reveal the long-term effects of applying metacognitive strategies in the geography classroom, on the other side. The limitation of the sample size due to the panel mortality raises the susceptibility to bias effects and lowers the validity [86] (p. 144). For reliable statements on the specific effectiveness of the interventions (inter-group-effects), according to G-Power, an increase of the sample size to n = 280 is required, under the assumption of small effect sizes and elimination of time effects [84] (p. 210). The interconnectedness of multi-layered aspects of metacognition obstructs the isolated comprehension of sub-processes and thus the isolated measurement and evaluation [11].

What aspect of “metacompetence” [109] (pp. 4–6) is precisely promoted by the applied methods, can therefore not be assessed, and demands further research. The effect also remains unclear of the interventions on the change of present (internal) causal models in the students’ mental lexicon for dealing with future problems [43,47], i.e., if thinking in causalities has generally changed. What is more, the aspect of an individual matching of the interventions according to personality, learner type, and working principles of students ought to be taken into account when further developing the interventions [110] (pp. 4–8), [111] (pp. 4–9), [112] (p. 341). Whether the theoretically deduced, open, explorative task type and its formal structure did have an effective influence on the development of complex geographical causal structures also ought to be empirically tested [102]. 

## 7. Conclusions

**Figure 7:**
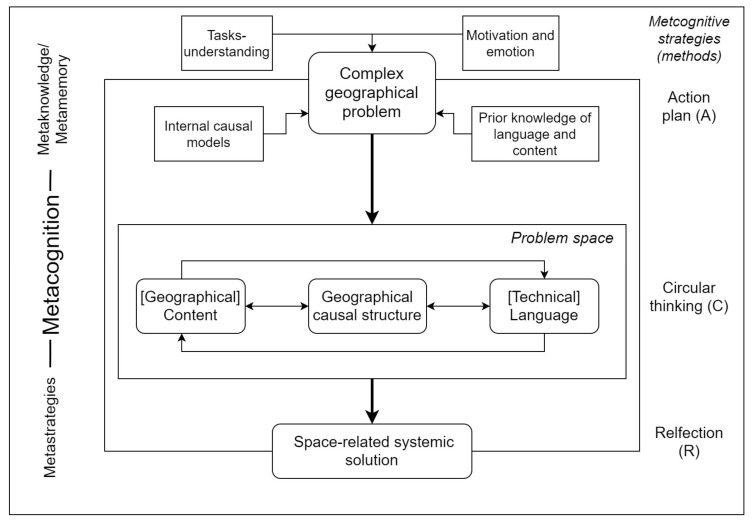
Content, linguistic and strategic impact of metacognition in geography classrooms.

The present study underlines that the application of metacognitive strategies in the context of complex problem-solving processes may be of great additional value for overcoming linear, monocausal thinking and developing a “multiperspective, systemic, and problem–solution-oriented thinking” [1] (p. 6) in the geography classroom. For this purpose, it is vital to train students in dealing with complex problems and therefore to continuously apply metacognitive strategies (Figure 7). Metacognitive methods promote students’ metacognitive skills (metaknowledge and metastrategies). They therefore reduce the technical, linguistic, and strategic barriers occurring when developing geographical causal structures (Figure 7). At the same time, metacognitive methods augment the understanding of system properties and system behavior and enable students to better detect space-related systemic solutions for complex problems within human–environment relations.

Teaching culture, however, can only change through alternative forms of measuring and assessing performance [113] (pp. 80–83). Yet another obstacle is posed by the task formats common with German school-leaving exams, which predominantly entail analytical problem (-solving) tasks. They demand, rather, schematic procedures (allocation of problems and problem categories) or routine actions scripts (pre-trained, material-based analysis and discussion; [60] (p. 15–16)). 

Open, complex problems [38] within creative tasks that require systemic competences and metacognitive thinking are missing, though. Moreover, the modelling of internal causal models by students through analytically dealing with problems is hardly possible within lessons [43] (p. 310). To alter mental models on causality, geographical causal structures need to be experienced in a slowed manner, in various shapes and graphical representations, interactively, reflexively, and playfully, the target being the augmented acquisition of systemic competences [24], [39] (pp. 7–8). 

A particular role in promoting systemic, multicausal thinking competences is given to the construction of learning tasks and the creation of instructional stages [114]. This begins with task comprehension and concerns aspects such as task duration, informatory range, used operators, as well as understanding and knowing technical terms, and providing scaffolding offered, e.g., reformulation of the task or visualizations. The activation of contentual and linguistic prior knowledge also needs to be given extended consideration. 

Apart from further empirical examination and conceptualization of the methods mentioned above, metacognitive support strategies for (de-) constructing complexity aiming at teaching multicausal, systemic thinking strategies, must be continued to, and as this study suggests, more often, be applied in the classroom and at the same time be scientifically elaborated.

## Figures and Tables

**Table 1:** Learning- and developmental-psychological, geographical, and linguistic barriers to developing multicausal geographical causal structures.

Barriers	Causes and Hindrances of (Multi-) Causal Linking	References
learning- and developmental-psychological	effort-avoiding adhesion to routine thinking operations (system 1) based on low motivation and emotions as a consequence of too little meaning individually attributed	[40,41,42]
	lack of causal knowledge or flawed, or rather, marginally developed, causal models	[46,47]
	age-dependent ability of spatial imagination and perception of causality	[48,49]
	concepts of causality depend on a specific social and cultural context	[50]
	missing feedback or flawed knowledge acquisition lead to flawed application of knowledge, i.e., the constructions of causal links as a consequence of a problem-solving process excessive demand due to coincidence and feedback effects of system variables and the causal relation	[51]
geographical	Overload due to coincidence and interaction effects of system variables and causal relations	[52]
	through emotional and affective loading as a result of sensual perception concepts of causality are determined by the close surrounding	[12,39]
	domain specific (technical) parameters of systems are not identified and impede resolving the relation types/effect relations of a system	[30]
linguistic	lack of linguistic resources leads to less contentual understanding, making thinking in complex interdependencies or causal effect relations less likely	[53,54]
	flawed recognition of system variables (technical terms) and flawed selection of verbs for expressing spatio-temporal causal links	[7,8]
	flawed understanding and knowledge of linguistic causal markers on the word and sentence level	[55,56]

**Table 3:** Number, topical matching, accuracy of the geographical causal structures as formulated by students in the intervention and control group before (t_0_) and after (t_1_) the application of metacognitive strategies.

Survey Groups	Total Number of Causal Links		Number of Topically Matching Links		Number of Correct Causal Links		Portion of Correct Links in %	
	t_0_	t_1_	t_0_	t_1_	t_0_	t_1_	t_0_	t_1_
	*M*	*M*	*M*	*M*	*M*	*M*	*M*	*M*
Intervention action plan	5.38	5.92	4.69	5.62	3.77	5.38	65.75	86.59
Intervention circular thinking	5.46	5.85	4.38	5.62	3.31	5.23	70.11	88.19
Intervention reflection	6.44	6.22	5.78	6.00	5.00	5.44	73.06	86.11
Control group	4.36	4.79	3.93	4.64	2.86	2.93	66.57	63.66

**Table 4:** Effectiveness of interventions on the development of mono- or multicausal geographical causal structures.

Survey Groups	Number of the Correct Monocausal Links			Number of the Correct Multicausal Link		
	t_0_	t_1_	∆t	t_0_	t_1_	∆t
	*M*	*M*	in %	*M*	*M*	in %
Control group	0.71	0.71	0.00%	2.14	2.43	13.55%
Intervention action plan	1.15	1.23	6.96%	2.54	4.23	66.54%
Intervention circular thinking	1.38	1.15	−16.67%	2.31	4.15	79.65%
Intervention reflection	2.89	0.78	−73.01%	2	4.67	133.50%

## Data Availability

The data presented in this study are available on request from the corresponding author. The data are not publicly available due to data protection and privacy.
